# Preliminary investigation of gut microbiota and associated metabolic pathways in the pathogenesis of primary central nervous system lymphoma

**DOI:** 10.3389/fonc.2025.1548146

**Published:** 2025-04-02

**Authors:** Zhuang Kang, Rong Zhang, Shenglan Li, Jiachen Wang, Mengqian Huang, Wenbin Li

**Affiliations:** Department of Neuro-Oncology, Cancer Center, Beijing Tiantan Hospital, Capital Medical University, Beijing, China

**Keywords:** gut microbiota, metabolomic analysis, metagenomics analysis, pathogenesis, primary central nervous system lymphoma (PCNSL)

## Abstract

**Background:**

Primary central nervous system lymphoma (PCNSL) is a rare and highly aggressive form of non-Hodgkin lymphoma, primarily confined to the central nervous system. In recent years, growing evidence has indicated that dysbiosis of the gut microbiota is closely associated with the development of various malignancies. This study aims to systematically explore the potential role of gut microbiota and their metabolic pathways in the pathogenesis of PCNSL by integrating metagenomic and metabolomic approaches.

**Materials and methods:**

A total of 33 PCNSL patients and 32 healthy controls were enrolled in this study, and fecal samples were collected from each participant. The fecal samples were analyzed using metagenomic and metabolomic techniques, followed by KEGG pathway enrichment analysis to investigate the biological pathways enriched by the differential gut microbiota and metabolites.

**Results:**

Significant differences were observed in the composition of gut microbiota and metabolites between PCNSL patients and healthy controls. In the gut microbiota of PCNSL patients, the abundance of the phylum Proteobacteria was markedly increased, while the *Firmicutes*/*Bacteroidetes* (*F/B*) ratio was significantly elevated. Metabolomic analysis revealed that the abundance of oleamide was significantly reduced in the PCNSL group, while the relative abundance of deoxycholic acid was significantly elevated. KEGG pathway analysis indicated that the differential gut microbiota and metabolites were primarily involved in key metabolic pathways such as nitrogen metabolism, phenylalanine metabolism, purine metabolism, and pyrimidine metabolism, with these pathways being more active in PCNSL patients.

**Conclusion:**

This study is the first to systematically investigate the differences in gut microbiota and their metabolites between PCNSL patients and healthy individuals, highlighting the potential role of gut microbiota alterations in the pathogenesis of PCNSL.

## 1 Introduction

Primary central nervous system lymphoma (PCNSL) is a rare and aggressive subtype of peripheral non-Hodgkin lymphoma (NHL), primarily affecting the brain, eyes, and spinal cord (1, 2). Treatment strategies typically include chemotherapy, radiotherapy, and stem cell transplantation (3). Although PCNSL represents a small proportion of all brain tumors, it has gained significant attention due to its poor prognosis, with a 5-year survival rate of only 30%-40% (1, 2). PCNSL typically occurs in immunocompromised individuals, such as those with HIV/AIDS or organ transplant recipients, though it can also affect immunocompetent patients (4–7). Current research suggested that the pathogenesis of PCNSL involved multiple factors, including genetic mutations, immune dysregulation, and the tumor microenvironment (5–7). Despite aggressive treatment, the survival rate for PCNSL remains low, highlighting the need for a deeper understanding of its molecular mechanisms and novel therapeutic approaches. In recent years, the potential role of the gut microbiota in modulating brain tumor development has garnered considerable attention, offering new avenues for therapeutic intervention.

The microbial communities present in the gastrointestinal ecosystem are referred to as gut microbiota, which play a crucial role in maintaining the intestinal mucosal barrier, immune homeostasis, and metabolic balance (8, 9). Humans share a symbiotic relationship with their gut microbiota, and dysbiosis has been recognized as a significant factor in the development of various diseases, including tumors (10, 11). Studies have indicated that gut microbiota dysbiosis may contribute to the occurrence of solid tumors such as gastric cancer, colorectal cancer, cholangiocarcinoma, hepatocellular carcinoma, and breast cancer (12, 13). Several studies have also demonstrated gut microbiota dysbiosis in lymphoma patients (14, 15). The gut-brain axis refers to the direct and indirect interactions between the gut microbiota and their metabolites with various cellular components of the central nervous system through immune signaling, such as metabolite-sensing receptors and the cannabinoid pathway (11, 16). With the discovery of the gut-brain axis, gut microbes are also thought to potentially play a role in the pathogenesis of brain tumors (11, 17). Previous research has indicated that gut microbiota is closely associated with the growth of various brain tumors, such as gliomas (11, 18) and meningiomas (18). Although some studies have explored the diversity of gut microbiota in PCNSL patients and healthy controls, to our knowledge, no studies have yet integrated metagenomic and metabolomic analyses to investigate the role of gut microbiota in the pathogenesis of PCNSL.

Compared to genomics and proteomics, metagenomics allows for the direct extraction and analysis of the genomes of all microorganisms from environmental samples, thereby capturing metabolic changes in tumor cells more effectively (19). As an effective tool for quantifying the composition of gut microbiota, metagenomics has been widely applied to investigate the relationships between gut microbiota and cancers, inflammatory diseases, and metabolic disorders (20). Metabolomics, on the other hand, reflects the metabolic state of an organism by characterizing changes in metabolites, revealing metabolic features of diseases and potential biomarkers. By integrating metagenomic and metabolomic analyses, researchers can explore the interactions between microbial communities and host metabolism in depth, making it particularly suitable for studying complex diseases such as cancer. This combined approach provides a holistic perspective from both ecological and metabolic viewpoints, aiding in the identification of novel biomarkers and therapeutic targets.

In this study, we performed a comprehensive analysis combining metagenomic sequencing and metabolomics on fecal samples to identify differences in gut microbiota and metabolites between the patient group and the control group. Through pathway enrichment analysis, we further elucidated the relevant metabolic pathways to clarify the potential role of gut microbiota in the pathogenesis of primary central nervous system lymphoma.

## 2 Materials and methods

### 2.1 Study population selection

This study included a total of 65 participants, comprising 33 patients with primary central nervous system lymphoma and 32 healthy controls. Participants with a history of severe gastrointestinal diseases or long-term antibiotic use were excluded from both groups. All PCNSL patient samples were collected after a definitive pathological diagnosis post-surgery, before the initiation of any subsequent treatments. Detailed clinical information of the PCNSL patient group and healthy control group was shown in **Supplementary Table S1**. All participants provided informed consent prior to enrollment. The PCNSL patients were recruited from the Neuro-Oncology Comprehensive Treatment Unit of Beijing Tiantan Hospital, Capital Medical University. All patients were diagnosed through stereotactic biopsy prior to sampling and had not received any chemotherapy or targeted therapy post-surgery. Healthy controls were excluded if they had a history of severe gastrointestinal diseases, long-term medication use, or regular consumption of probiotic products.

### 2.2 Metagenomic analysis

#### 2.2.1 DNA extraction of environmental microorganisms using CTAB method

Add 1000 µL of CTAB lysis buffer to a 2.0 mL Eppendorf tube, followed by an appropriate amount of lysozyme. Then, introduce the sample into the lysis buffer. Place the tube in a water bath at 65°C, gently inverting the tube several times to ensure thorough lysis of the sample. After lysis, centrifuge the sample to collect the supernatant. Add a mixture of phenol (pH 8.0): chloroform: isoamyl alcohol (25:24:1) to the supernatant, invert the tube thoroughly, and centrifuge at 12,000 rpm for 10 minutes.

Collect the upper aqueous phase, then add chloroform: isoamyl alcohol (24:1), invert the tube again, and centrifuge at 12,000 rpm for 10 minutes. Transfer the supernatant to a 1.5 mL centrifuge tube, add an equal volume of isopropanol, gently invert to mix, and incubate at -20°C for precipitation. Centrifuge at 12,000 rpm for 10 minutes, carefully discard the supernatant without disturbing the pellet. Wash the pellet twice with 1 mL of 75% ethanol, retaining a small amount of liquid for further centrifugation to collect the pellet, and use a pipette to aspirate the residual liquid. Dry the pellet in a biosafety cabinet or allow it to air dry at room temperature. Resuspend the DNA pellet in an appropriate volume of ddH_2_O. If necessary, incubate at 55-60°C for 10 minutes to facilitate dissolution. Add 1 µL of RNase A and incubate at 37°C for 15 minutes to digest RNA present in the sample.

#### 2.2.2 Sample detection

DNA samples were assessed using the following three methods: (1) Agarose Gel Electrophoresis: This technique was employed to analyze the purity and integrity of the DNA samples. (2) Nanodrop Measurement: The OD 260/280 ratio was measured to evaluate the purity of the DNA. (3) Qubit 2.0 Fluorometer: This instrument was used for precise quantification of DNA concentration. Once the DNA samples passed quality checks, a Covaris ultrasonic sonicator was used to randomly fragment the DNA. Following fragmentation, the samples underwent a series of steps including end repair, addition of an A-tail, ligation of sequencing adapters, purification, and PCR amplification to complete the library preparation. After constructing the library, initial quantification was performed using Qubit 2.0, and the library was diluted accordingly. Subsequently, the Agilent 2100 was used to assess the size of the insert fragments, ensuring they met the expected specifications. Finally, Q-PCR was utilized to accurately determine the effective concentration of the library, confirming that the library quality met the sequencing requirements.

#### 2.2.3 Sequencing

After passing library quality control, libraries were pooled in proportion to their effective concentration and the target output data, and then loaded onto the flow cell. Cluster generation was performed using cBOT, followed by high-throughput sequencing on the Illumina PE150 platform (2x150 paired-end sequencing). This process yielded metagenomic sequences from bacteria, fungi, and viruses present in fecal samples. Raw data were subjected to quality control using KneadData software (based on Trimmomatic) for data filtering. Host sequences were removed with Bowtie2. Species identification was performed using Kraken2, and species annotation was done using a custom-built microbial database. The Bracken software was used to predict the actual relative abundance of species in the samples. For functional annotation, HUMAnN2 software (based on the DIAMOND algorithm) was used to map reads from each sample to the UniRef90 database, generating functional annotations and relative abundance tables for various databases. Abundance clustering analysis, principal coordinate analysis (PCoA), and non-metric multidimensional scaling (NMDS, for species only) were performed based on the abundance tables of species and functions, along with sample clustering analysis. For data that included group information, linear discriminant analysis effect size (LEfSe) biomarker analysis and Dunn’s test were conducted to explore differences in species and functional composition between samples, revealing potential biological significance.

### 2.3 Metabolomics analysis

#### 2.3.1 Metabolite extraction

##### 2.3.1.1 Tissue samples

For tissue samples, 100 mg of each tissue was weighed and fully ground in liquid nitrogen. The tissue was then resuspended in pre-cooled 80% methanol, followed by vigorous vortex mixing. After incubating the samples on ice for 5 minutes, they were centrifuged at 15,000 g for 20 minutes at 4°C. A portion of the supernatant was diluted with LC-MS-grade water to achieve a final concentration of 53% methanol. The samples were transferred to new Eppendorf tubes and centrifuged again at 15,000 g for 20 minutes at 4°C. Finally, the supernatant was collected and injected into the LC-MS/MS system for analysis (21).

##### 2.3.1.2 Liquid samples

For liquid samples, 100 μL of the sample was placed in an EP tube, resuspended in pre-cooled 80% methanol, and thoroughly vortexed. The sample was incubated on ice for 5 minutes and then centrifuged at 15,000 g for 20 minutes at 4°C. A portion of the supernatant was diluted with LC-MS-grade water to a final concentration of 53% methanol. The sample was transferred to a new Eppendorf tube and centrifuged again at 15,000 g for 20 minutes at 4°C. Finally, the supernatant was collected and injected into the LC-MS/MS system for analysis (22, 23).

##### 2.3.1.3 Cell or bacterial samples

For cell or bacterial samples, they were placed in an EP tube, resuspended in pre-cooled 80% methanol, and vortexed thoroughly. After thawing the sample on ice and vortexing for 30 seconds, it was subjected to 6 minutes of ultrasonication. The sample was then centrifuged at 5,000 rpm for 1 minute at 4°C. The supernatant was collected and freeze-dried, followed by resuspension in 10% methanol. Finally, the solution was injected into the LC-MS/MS system for analysis (24, 25).

##### 2.3.1.4 Cell or bacterial culture medium samples

For culture medium samples, 1 mL of the medium was freeze-dried and resuspended in pre-cooled 80% methanol, followed by thorough vortexing. The sample was incubated on ice for 5 minutes and centrifuged at 15,000 g for 15 minutes at 4°C. A portion of the supernatant was diluted with LC-MS-grade water to a final concentration of 53% methanol. The sample was transferred to a new Eppendorf tube and centrifuged again at 15,000 g for 15 minutes at 4°C. The supernatant was then collected and injected into the LC-MS/MS system for analysis. This completes the extraction procedure for different sample types prior to LC-MS/MS analysis.

#### 2.3.2 UHPLC-MS/MS analysis

Ultra-high-performance liquid chromatography-tandem mass spectrometry (UHPLC-MS/MS) analysis was performed using a Vanquish UHPLC system (Thermo Fisher, Germany) coupled with an Orbitrap Q Exactive™ HF mass spectrometer. Samples were separated over a 17-minute linear gradient on a Hypesil Gold column (100×2.1 mm, 1.9 μm) at a flow rate of 0.2 mL/min. In positive ion mode, the mobile phase A consisted of 0.1% formic acid in water, and mobile phase B was methanol. In negative ion mode, mobile phase A was 5 mM ammonium acetate (pH 9.0), and mobile phase B was methanol. The solvent gradient was programmed as follows: initial 2% B for 1.5 minutes; gradient ramp from 2% to 100% B over 3 minutes; holding at 100% B for 10 minutes; then returning rapidly to 2% B at 10.1 minutes and maintaining it until 12 minutes.

Mass spectrometry was operated in both positive and negative ionization modes with a spray voltage of 3.5 kV, a capillary temperature of 320°C, sheath gas flow at 35 psi, auxiliary gas flow at 10 L/min, an S-lens RF level set to 60, and an auxiliary gas heater temperature of 350°C. This method ensured high sensitivity and accuracy, making it suitable for quantitative analysis in metabolomics and other biomarker studies.

#### 2.3.3 Data processing and metabolite identification

The raw data files generated by UHPLC-MS/MS were processed using Compound Discoverer 3.1 (CD3.1, ThermoFisher) software, which includes peak alignment, peak extraction, and metabolite quantification. The main parameters were set as follows: retention time tolerance of 0.2 minutes, mass accuracy tolerance of 5 ppm, signal intensity tolerance of 30%, signal-to-noise ratio set to 3, and minimum intensity threshold. Peak intensities were normalized to the total ion current, and the normalized data were used to deduce molecular formulas through the analysis of adduct ions, molecular ions, and fragment ions.

Metabolite identification was performed by matching the data with the mzCloud (https://www.mzcloud.org/), mzVault, and MassList databases, ensuring precise qualitative and relative quantitative results. This data processing workflow guarantees high-resolution metabolite identification, making it suitable for biomarker discovery and biochemical pathway analysis in metabolomics research.

#### 2.3.4 Data analysis

Metabolite annotation was performed using the Kyoto Encyclopedia of Genes and Genomes (KEGG) database (https://www.genome.jp/kegg/), HMDB database (https://hmdb.ca/metabolites), and LIPIDMaps database (http://www.lipidmaps.org/). Functional pathway enrichment analysis was performed to reveal the differential functional pathways between the two groups. Data normalization, principal component analysis (PCA), partial least squares discriminant analysis (PLS-DA), orthogonal PLS-DA (OPLS-DA), random forest analysis (RF), and support vector machine (SVM) analysis were all carried out using the MetaboAnalystR package in R software (26). To ensure the data conformed to a normal distribution, normalization was performed using the Normalization function in MetaboAnalystR, with parameters such as MedianNorm, LogNorm, and AutoNorm selected accordingly. Univariate analysis (t-test) was used to calculate statistical significance (P-value). After normalizing the data to z-scores, clustering heatmaps were generated using the Pheatmap package in R. Additionally, volcano plots were constructed using the ggplot2 package in R, plotting log2 (Fold Change) against -log10 (P-value) to identify significantly altered metabolites. This comprehensive data analysis pipeline provides robust support for the identification of key metabolites and the evaluation of statistical significance in metabolomics research.

### 2.4 Statistical analysis

All bioinformatics data analyses were performed using R software (version 4.1.1) in combination with Bioconductor packages. Laboratory data analyses were conducted using GraphPad Prism software. Statistical significance was defined by the following criteria: Spearman correlation coefficient |R| > 0.3, variable importance in projection (VIP) score > 1, log2 (Fold Change) > 1, and P-value < 0.05. Results meeting these thresholds were considered statistically significant, indicating potential biological relevance of the observed correlations or differences.

## 3 Results

### 3.1 Composition, species diversity, and phylum-level differences in gut microbiota

**Figure 1:**
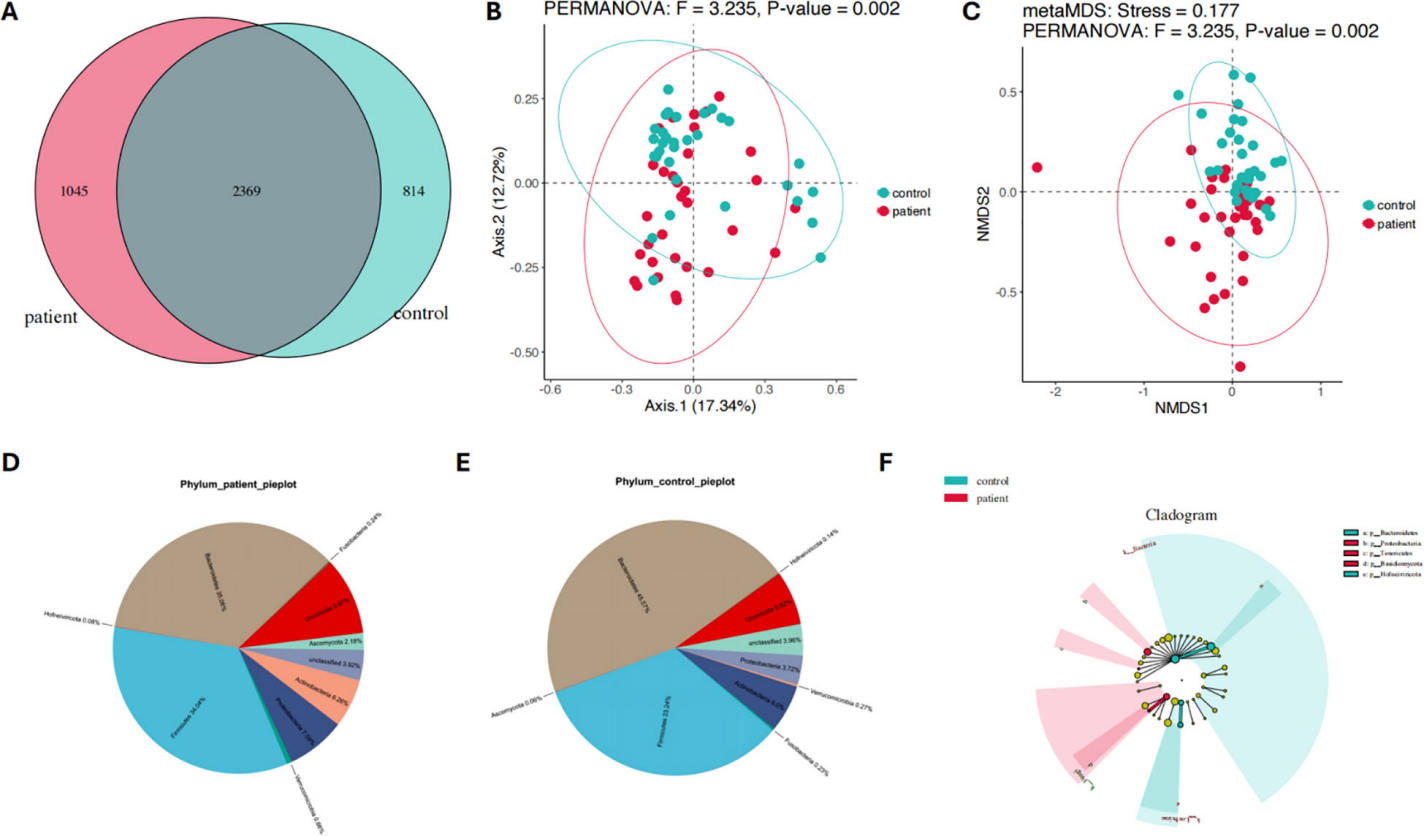
Composition and species diversity of the gut microbiota in the patient and control groups. **(A)** The Venn diagram illustrates the compositional features of the gut microbiota in the patient and control groups **(B)** PCoA analysis shows significant differences in the gut microbiota composition between the patient and control groups **(C)** NMDS analysis confirms the significant differences in the gut microbiota composition between the patient and control groups **(D)** The relative abundance distribution of the gut microbiota at the phylum level in the patient group **(E)** The relative abundance distribution of the gut microbiota at the phylum level in the control group **(F)** The differences of gut microbiota at the phylum level between the patient and control groups.

Firstly, the differences in gut microbiota composition between PCNSL patients and healthy controls were analyzed. A Venn diagram revealed that 2,369 gut microbial species were shared between the two groups (**Figure 1A**). In addition, 1,045 species were unique to the PCNSL group, while 814 species were unique to the control group, indicating a potential significant difference in gut microbiota composition between the two groups. PCoA further validated this difference, showing a significant variation in microbial composition between the two groups (p = 0.002, F = 3.235) (**Figure 1B**). Moreover, NMDS analysis also confirmed the significant divergence in gut microbiota between the groups (p = 0.002, F = 3.235, Stress = 0.177) (**Figure 1C**). These findings suggest notable differences in gut microbial diversity and composition between PCNSL patients and healthy controls.

Pie charts were used to further illustrate the major gut microbiota composition and their relative abundance at the phylum level between the patient and control groups (**Figures 1D, E**) In the PCNSL patient group, the dominant phyla were *Bacteroidetes* (35.06%), *Firmicutes* (34.04%), *Uroviricota* (9.97%), *Proteobacteria* (7.59%), *Actinobacteria* (6.26%), *Ascomycota* (2.18%), *Verrucomicrobia* (0.66%), *Fusobacteria* (0.24%), and *Hofneiviricota* (0.08%) (**Figure 1D**). In contrast, the main phyla in the healthy control group included *Bacteroidetes* (45.57%), *Firmicutes* (33.24%), *Uroviricota* (6.82%), *Actinobacteria* (6.0%), *Proteobacteria* (3.72%), *Verrucomicrobia* (0.27%), *Fusobacteria* (0.23%), *Hofneiviricota* (0.14%), and *Ascomycota* (0.06%) (**Figure 1E**).

These findings indicate significant differences in the phylum-level microbiota composition between the two groups. To further explore these differences, linear discriminant analysis effect size (LEfSe) was applied (**Figure 1F**). The analysis revealed that *Basidiomycota* (p=0.0025), *Proteobacteria* (p=0.04995), and *Tenericutes* (p=0.0116) were significantly more abundant in the PCNSL patient group compared to the control group. On the other hand, *Hofneiviricota* (p=0.0296) and *Bacteroidetes* (p=0.0454) were more prevalent in the healthy controls. These results suggest distinct microbial profiles at the phylum level between PCNSL patients and healthy individuals.

### 3.2 Functional pathway enrichment analysis of differential gut microbiota

**Figure 2:**
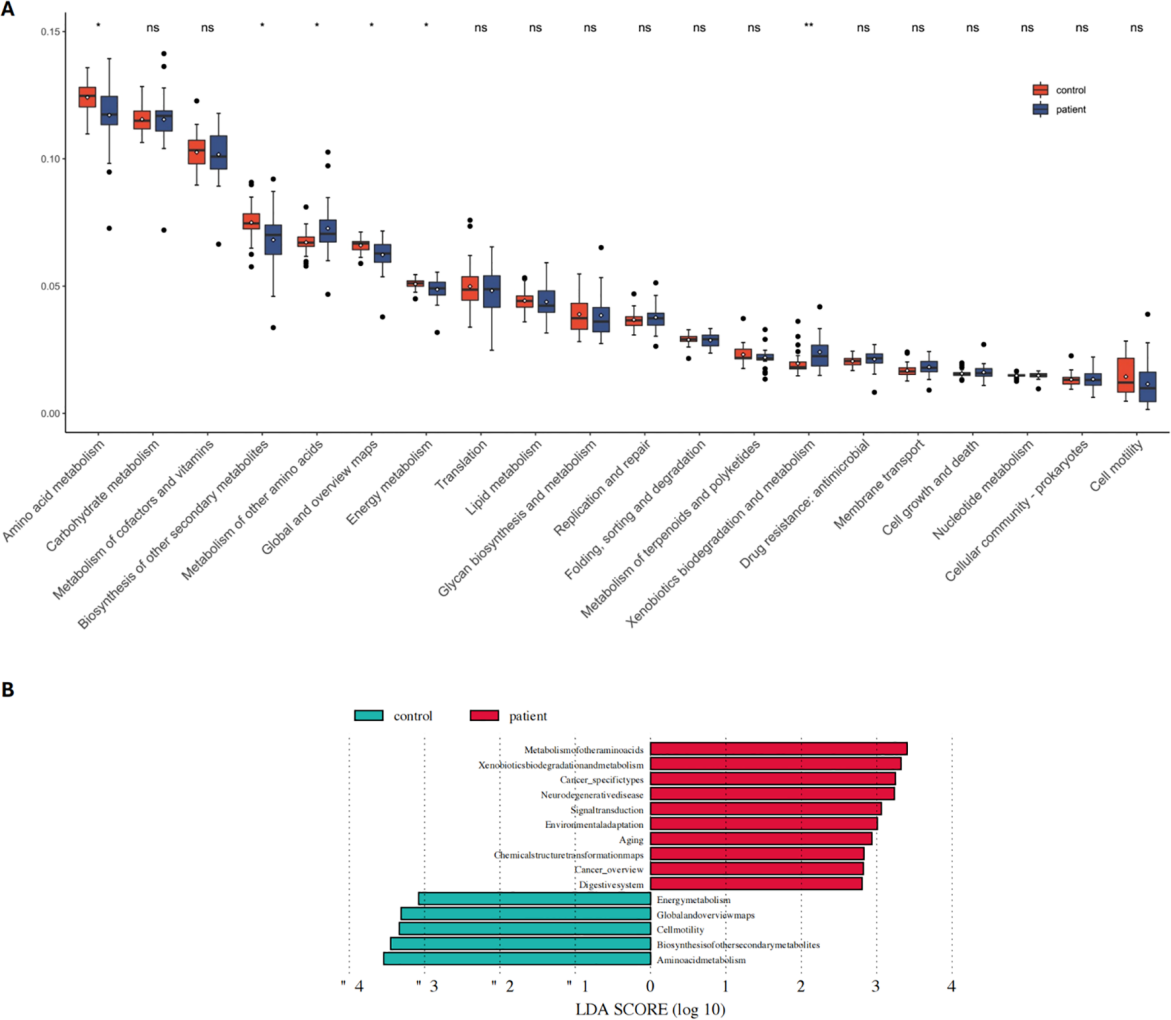
Functional pathway analysis related to differential microbes in the patient and control groups. **(A)** KEGG pathway analysis reveals significant differences in functional pathways between the patient and control groups **(B)** Comparison of dominant metabolic pathways between the patient and control groups. NS means no statistical significance. *P < 0.05, **P < 0.01.

Functional pathway enrichment analysis was conducted on the significantly different gut microbiota between the PCNSL patient group and the healthy control group using LEfSe and KEGG analysis. This aimed to further explore metabolic pathway differences between the two groups. KEGG analysis further revealed higher activity in the metabolism of other amino acids and xenobiotics biodegradation and metabolism in the PCNSL group. In contrast, pathways associated with amino acid metabolism, biosynthesis of other secondary metabolites, global and overview maps, and energy metabolism were more prominently expressed in the healthy control group (**Figure 2A**).

**Figure 2B** showed that pathways related to chemical structure transformation maps (p=0.0018), neurodegenerative diseases (p=0.0041), xenobiotics biodegradation and metabolism (p=0.0175), cancer-specific types (p=0.0181), and aging (p=0.0426) were significantly more active in the PCNSL group (**Figure 2B**).

Further analysis of these functional pathways showed that the metabolic activity of the D-alanine metabolism pathway was significantly higher in the PCNSL group compared to controls. On the other hand, pathways such as valine, leucine, and isoleucine biosynthesis, biosynthesis of amino acids, thiamine metabolism, biotin metabolism, and 2-oxocarboxylic acid metabolism exhibited higher metabolic activity in the control group (**Supplementary Figure S1**). These findings suggest that gut microbiota may contribute to the onset and progression of PCNSL by influencing these metabolic pathways.

### 3.3 Analysis of metabolite composition, differences, correlations, and characteristic metabolites

**Figure 3:**
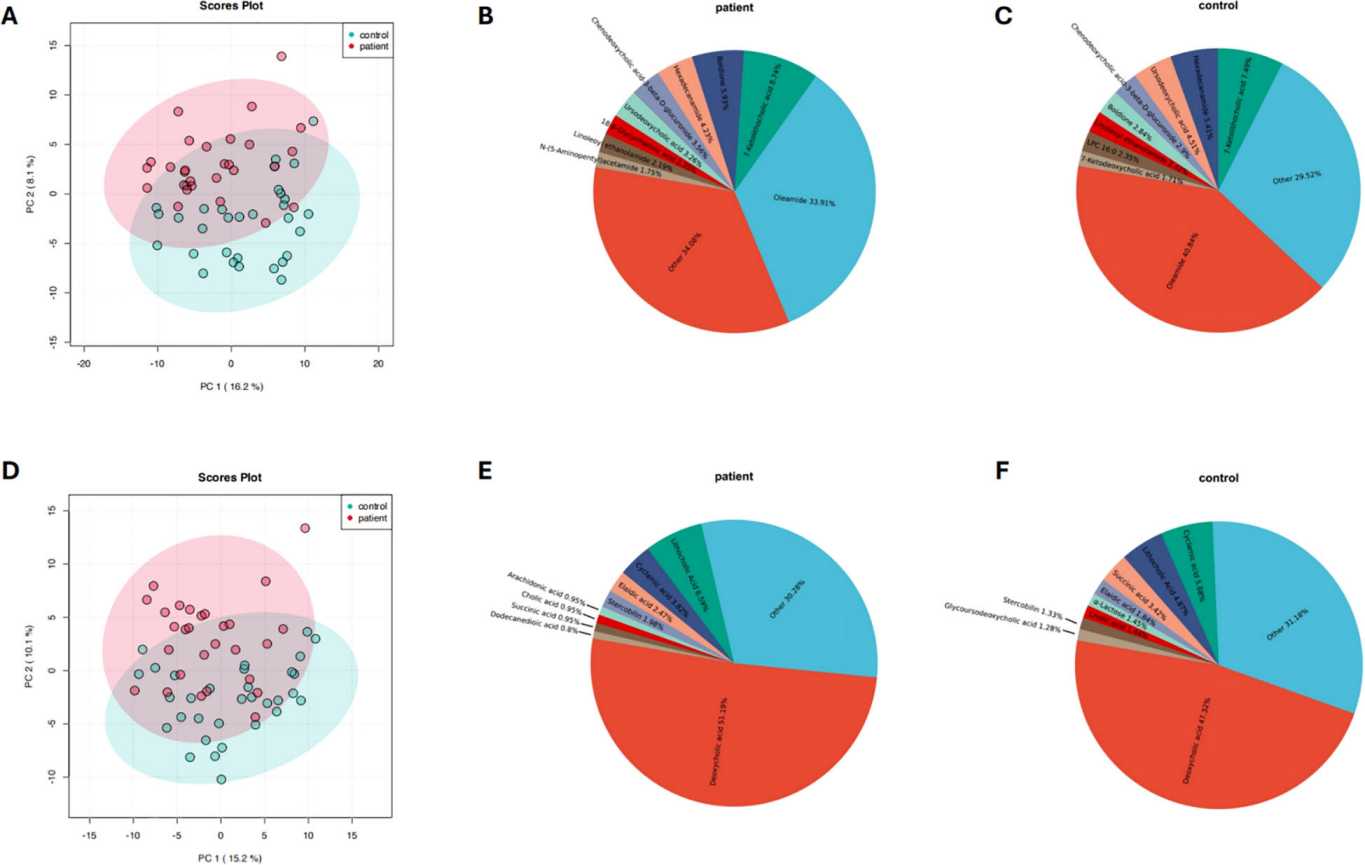
Composition and differences in positive and negative ion metabolites between the patient and control groups. **(A)** Analysis of differences in positive ion metabolites between the patient and control groups **(B)** Relative composition of positive ion metabolites in the patient group **(C)** Relative composition of positive ion metabolites in the control group **(D)** Analysis of differences in negative ion metabolites between the patient and control groups **(E)** Relative composition of negative ion metabolites in the patient group **(F)** Relative composition of negative ion metabolites in the control group.

Metabolomic analysis was performed to further investigate the differences in fecal metabolite composition between the PCNSL patient group and the healthy control group. Principal component analysis (PCA) revealed significant differences in the composition of positive ion metabolites between the two groups (**Figure 3A**). Pie chart analysis demonstrated that the dominant positive ion metabolites differed markedly between the groups, with the relative abundance of oleamide significantly lower in the PCNSL group compared to the control group (**Figures 3B, C**). Similarly, negative ion metabolites were analyzed, showing significant differences between the groups, particularly with a higher proportion of deoxycholic acid in the PCNSL group (**Figures 3D–F**).

In addition, a combination of machine learning techniques, support vector machine (SVM) analysis, and univariate analysis was applied to further investigate the differences in both positive and negative ion metabolites between the two groups. The results consistently indicated significant differences in metabolite composition, suggesting that these metabolites may serve as potential biomarkers for PCNSL (**Supplementary Figure S2**).

### 3.4 Pathway enrichment analysis of differential metabolites

**Figure 4:**
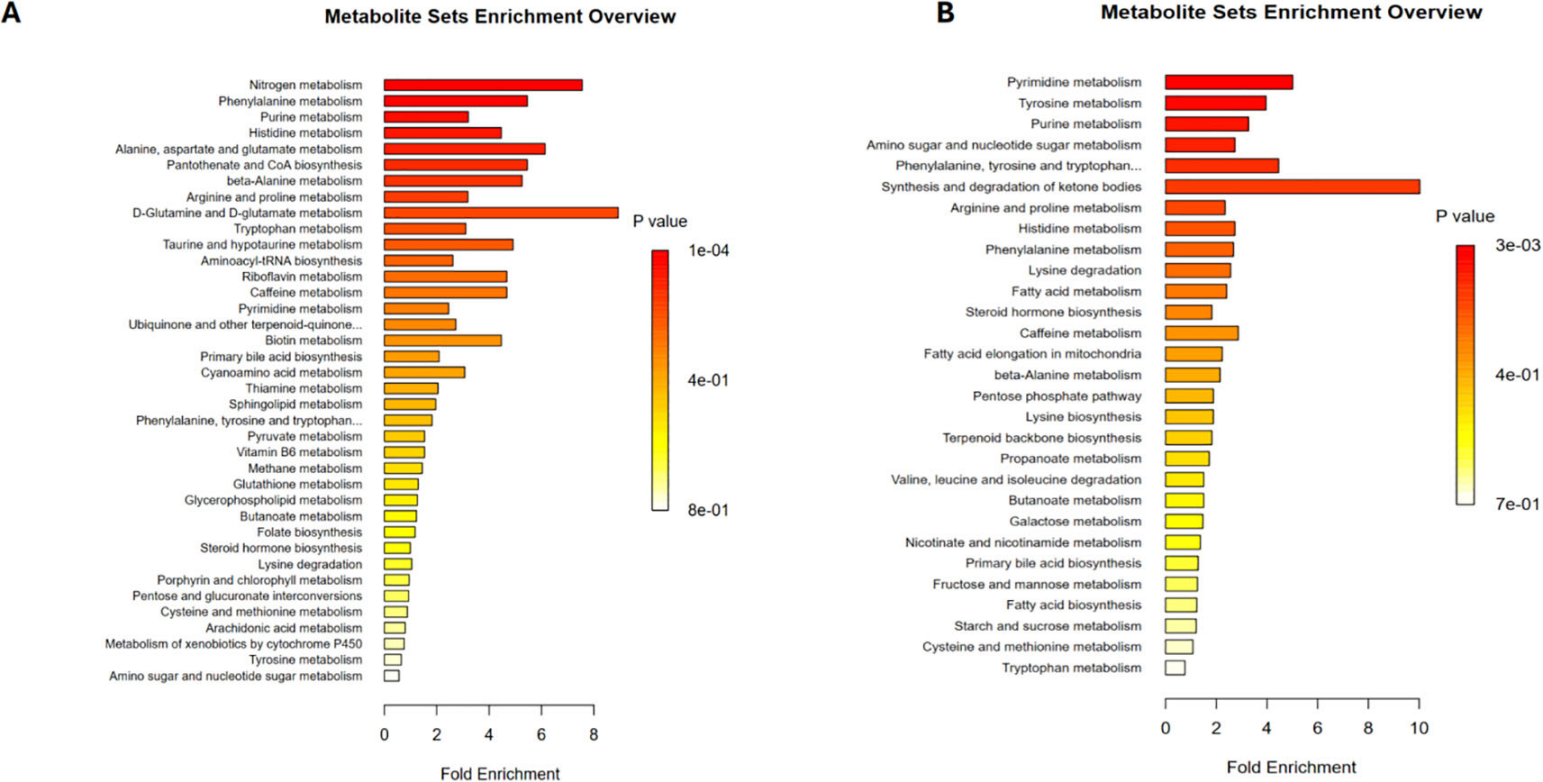
Metabolic pathway analysis related to differential metabolites. **(A)** Metabolic pathways associated with differential positive ion metabolites in the patient and control groups **(B)** Metabolic pathways associated with differential negative ion metabolites in the patient and control groups.

Lastly, a pathway enrichment analysis was conducted on the differential metabolites between the PCNSL patient group and the healthy control group to explore the metabolic pathways these metabolites might affect. The results revealed that the differential positive ion metabolites were mainly associated with the following metabolic pathways (**Figure 4A**): nitrogen metabolism, phenylalanine metabolism, purine metabolism, histidine metabolism, alanine, aspartate, and glutamate metabolism, pantothenate and CoA biosynthesis, beta-alanine metabolism, arginine and proline metabolism, D-glutamine and D-glutamate metabolism, and tryptophan metabolism.

For the differential negative ion metabolites, enrichment analysis indicated their involvement in pyrimidine metabolism, tyrosine metabolism, and purine metabolism (**Figure 4B**). These findings suggest that the differential metabolites may contribute to the pathogenesis and progression of PCNSL by regulating these metabolic pathways.

## 4 Discussion

Primary central nervous system lymphoma is a rare form of lymphoma primarily confined to the central nervous system. Due to its low incidence, current knowledge regarding PCNSL remains fragmented, and its pathogenesis has not been fully elucidated (27). Alterations in the gut microbiota may significantly influence host metabolism, inflammatory responses, and immune function (28). The gut microbiota communicates bidirectionally with the central nervous system through the gut-brain axis, and its dysregulation may contribute to the development of central nervous system diseases (29, 30). Therefore, our study focuses on the potential role of gut microbiota in the pathogenesis of PCNSL. Through a multi-level analysis of gut microbiota and metabolites, we identified significant differences between PCNSL patients and healthy controls in terms of microbial composition and metabolite profiles. This study provides novel insights into the potential involvement of gut microbiota in PCNSL, highlighting the role of microbiota and metabolites in the disease’s pathophysiology.

In terms of gut microbiota composition, Venn diagrams, PCoA, and NMDS analysis consistently demonstrated significant differences in microbial communities and species diversity between PCNSL patients and healthy controls. This finding is consistent with previous studies, such as the research by Louha et al., which demonstrated significant changes in microbial diversity in PCNSL patients (31). Notably, our study showed that the relative abundance of *Proteobacteria* was higher in the patient group. Previous studies have shown that an increase in *Proteobacteria* can promote cancer development through multiple mechanisms. Overgrowth of *Proteobacteria* impairs intestinal barrier function, increases gut permeability, and allows bacterial metabolites and inflammatory mediators to enter the bloodstream, triggering systemic inflammation. This creates a favorable environment for tumorigenesis (32, 33). Additionally, the upregulation of *Proteobacteria* may alter the tumor microenvironment through immune suppression and angiogenesis, enhancing tumor immune evasion and thus promoting tumor development and progression (34–37). Some *Proteobacteria* pathogens can also produce carcinogenic toxins directly linked to cancer promotion (35, 38).

Our findings also revealed a significantly higher *Firmicutes*/*Bacteroidetes* (*F/B*) ratio in the PCNSL patient group compared to the control group. The *F/B* ratio is widely recognized as a marker of gut dysbiosis, and changes in *Proteobacteria* are thought to be a major cause of this dysbiosis (39). Other bacterial phyla’s imbalances may not significantly impact the *F/B* ratio (39, 40). Previous studies have demonstrated a positive correlation between the *F/B* ratio, tumor burden, cell proliferation, and inflammatory cytokines, with an elevated *F/B* ratio being confirmed as a risk factor for colorectal cancer (39, 41). In this study, the significantly higher *F/B* ratio in PCNSL patients, along with increased *Proteobacteria* abundance, suggests that these patients experience pronounced gut dysbiosis. This dysbiosis may be closely related to the development of PCNSL, with gut microbiota potentially influencing the disease’s pathogenesis through metabolic and immune regulatory pathways.

In the metabolomic differential analysis, we found significant differences in both positive and negative ion metabolites between PCNSL patients and healthy controls, suggesting that these metabolites may play a crucial role in disease development. Among the positive ion metabolites, oleamide showed a significant decrease in abundance in the patient group, while deoxycholic acid was significantly elevated among the negative ion metabolites. Oleamide is a bioactive lipid that can be derived from endogenous synthesis (42) or produced by gut microbiota (43). After entering systemic circulation, it is excreted primarily through the hepatobiliary pathway (44, 45). Oleamide is believed to regulate processes such as cell proliferation, differentiation, and apoptosis through pathways related to the endocannabinoid system and G-protein-coupled receptors, thus influencing tumor growth (46–50). Furthermore, oleamide possesses anti-inflammatory properties, and studies have shown that it may reduce cancer risk by mitigating inflammation, thus inhibiting tumor progression. Oleamide also plays an immunomodulatory role in the tumor microenvironment, affecting tumor cell survival and migration. It has been reported that oleamide plays a critical role in the development of liver and breast cancers (51–55). On the other hand, deoxycholic acid, a secondary bile acid, is closely linked to changes in gut microbiota and inflammatory responses, potentially promoting cancer through pro-inflammatory and carcinogenic effects on the intestinal mucosa. Increased deoxycholic acid levels have been strongly associated with colorectal cancer development (56–58). Therefore, we speculate that the downregulation of oleamide and the upregulation of deoxycholic acid may similarly contribute to the pathogenesis of PCNSL. These metabolic alterations likely play roles in modulating tumor-promoting inflammation and immune responses, suggesting potential mechanisms through which gut microbiota and metabolites influence PCNSL progression.

In the pathway enrichment analysis, we found that the differential metabolites between PCNSL patients and healthy controls were mainly involved in key metabolic pathways such as nitrogen metabolism, phenylalanine metabolism, purine metabolism, and pyrimidine metabolism. Previous studies have shown that metabolic dysregulation supports tumor cell growth while enabling immune evasion, playing a crucial role in lymphoma development and progression (5). Interestingly, these pathways were notably more active in the PCNSL patient group, suggesting that they may influence the pathogenesis of PCNSL by regulating energy metabolism, amino acid metabolism, and immune function. Interestingly, while there is no current literature linking alanine metabolism specifically to PCNSL, our findings suggest a potential mechanism where increased alanine metabolism may provide more pyruvate for gluconeogenesis, producing large amounts of glucose to support the rapid proliferation of tumor cells (59, 60). Alanine is also involved in the tricarboxylic acid (TCA) cycle, which is crucial for energy production. Previous studies have shown that in pancreatic ductal adenocarcinoma, alanine contributes carbon to the TCA cycle, allowing glucose to be used for nucleic acid synthesis, thereby promoting tumor growth (61, 62). Elevated levels of alanine metabolism have also been confirmed in melanoma and prostate cancer (60, 63). Thus, our study is the first to report changes in alanine metabolism in PCNSL patients. However, the precise role of these metabolic pathways in PCNSL requires further investigation. These findings provide a foundation for exploring how alanine metabolism and related pathways contribute to tumorigenesis in PCNSL, potentially revealing new therapeutic targets.

This study also has several limitations. First, the findings are based on the analysis of clinical fecal samples. While we have provided preliminary evidence suggesting that the gut microbiota may be involved in the pathogenesis of PCNSL, this research is still in the exploratory phase. We have not yet performed an in-depth interactive analysis of gut microbiota and metabolites, and therefore not yet comprehensively and systematically validated the specific mechanisms by which the gut microbiota may influence PCNSL. Future large-scale clinical studies and experimental validation are required to better understand its potential role in PCNSL. Secondly, although we have made efforts to control for various variables in the study, eliminating all potential bias related to the gut microbiota remains a challenge. The composition and function of the gut microbiota are influenced by various factors such as diet, antibiotic use, and the host immune status, which can differ significantly between individuals. Therefore, it is crucial to consider these potential confounders, and future research should further refine experimental designs to minimize bias and enhance the robustness of the findings.

## 5 Conclusion

In summary, this study demonstrates significant alterations in both the gut microbiota and metabolome of PCNSL patients, suggesting that these changes may contribute to the pathogenesis and progression of PCNSL through their involvement in multiple metabolic pathways. Future research should focus on further investigating these characteristic metabolites and microbial species, as well as their interactions with metabolic pathways. This will help to elucidate the underlying mechanisms of PCNSL development and provide a theoretical basis for the development of novel therapeutic strategies.

## Data Availability

The original contributions presented in the study are included in the article/**Supplementary Material**. Further inquiries can be directed to the corresponding author.
