# Supplementary figures and images for: Preliminary investigation of gut microbiota and associated metabolic pathways in the pathogenesis of primary central nervous system lymphoma
Source: Front Oncol. 2025 Apr 2;15:1548146. doi: 10.3389/fonc.2025.1548146 (PMC12000031; doi:10.3389/fonc.2025.1548146)

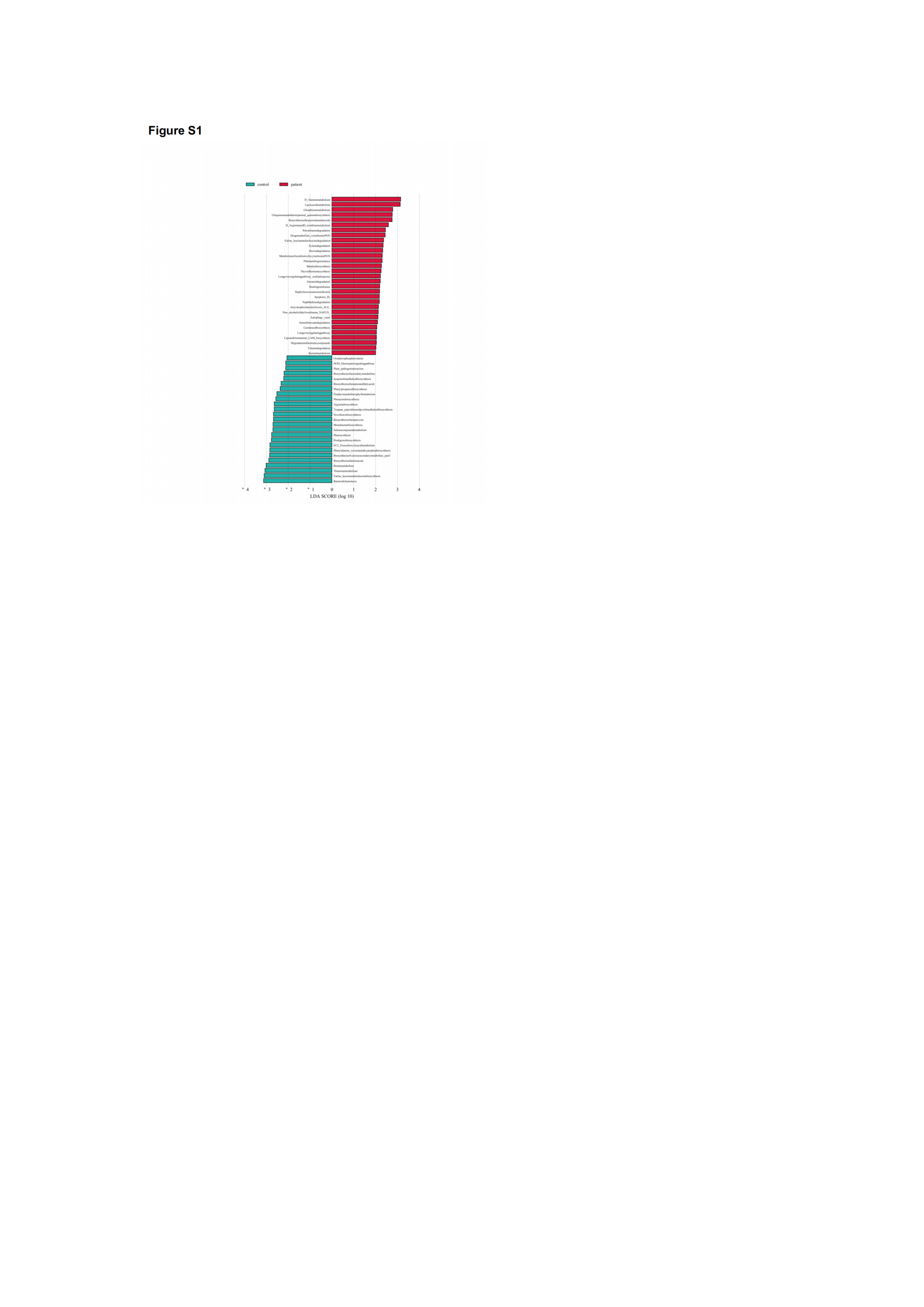

Supplement: Supplementary file 1 [file Image1.tif]

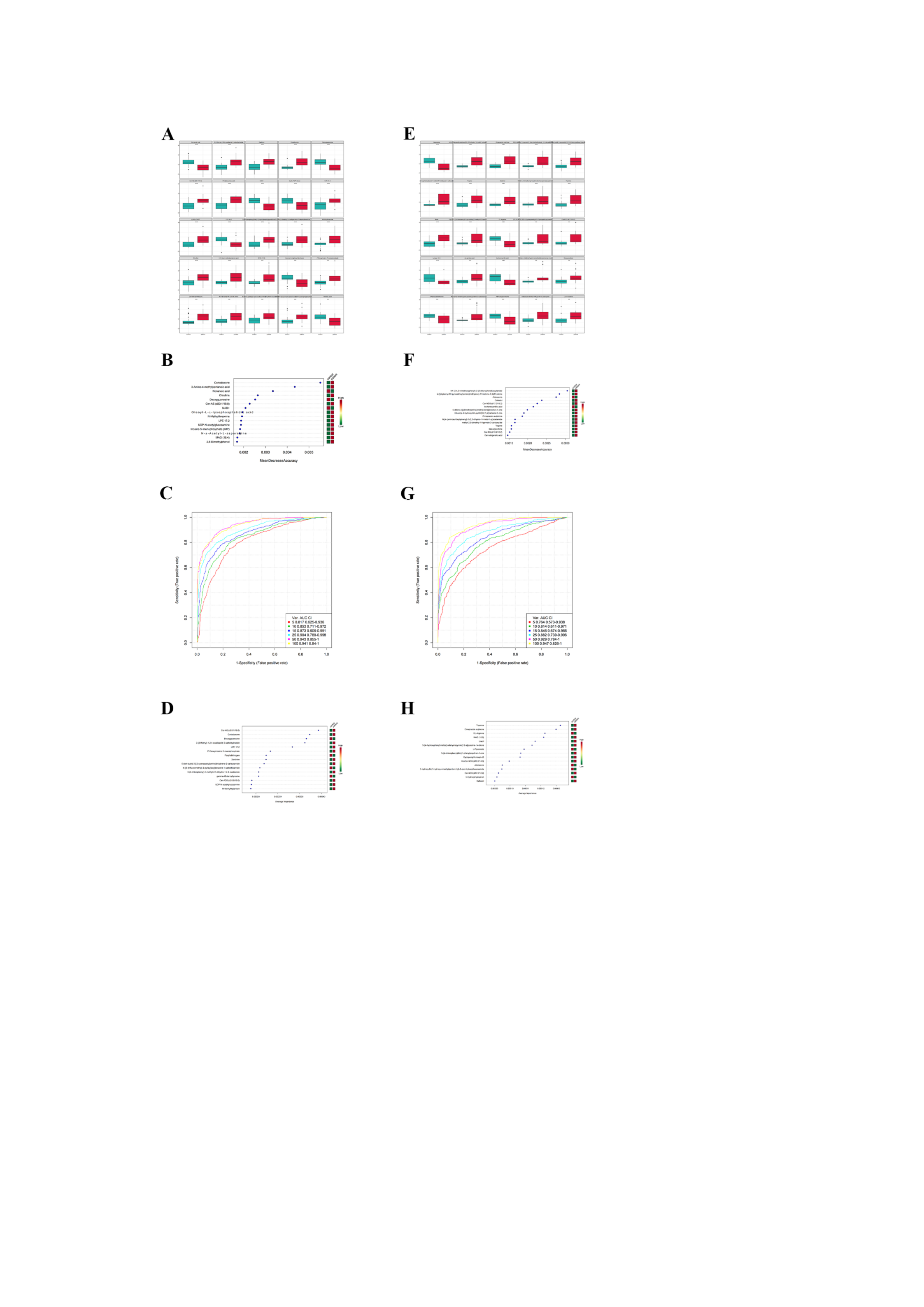

Supplement: Supplementary file 2 [file Image2.tif]
