# Supplementary material for: Preliminary investigation of gut microbiota and associated metabolic pathways in the pathogenesis of primary central nervous system lymphoma
Source: Front Oncol. 2025 Apr 2;15:1548146. doi: 10.3389/fonc.2025.1548146 (PMC12000031; doi:10.3389/fonc.2025.1548146)
Supplement: Supplementary file 3 [file Table1.docx]

| Table S1. Clinical Information of the PCNSL Patient Group and Healthy Control Group | | | | | | | | |
| --- | --- | --- | --- | --- | --- | --- | --- | --- |
| Group | ID | Age | Gender | Histopathology | Molecular Pathology | Treatment | Gastrointestinal Diseases | Long-term Antibiotic Use |
| PCNSL patients | P001 | 67 | Female | Diffuse Large B-Cell Lymphoma | CD20(+) | surgical resection | None | None |
| PCNSL patients | P002 | 62 | Male | Diffuse Large B-Cell Lymphoma | CD20(+) | stereotactic biopsy | None | None |
| PCNSL patients | P003 | 48 | Male | Diffuse Large B-Cell Lymphoma | CD20(+) | surgical resection | None | None |
| PCNSL patients | P004 | 68 | Male | Diffuse Large B-Cell Lymphoma | CD20(+) | stereotactic biopsy | None | None |
| PCNSL patients | P005 | 56 | Female | Diffuse Large B-Cell Lymphoma | CD20(+) | stereotactic biopsy | None | None |
| PCNSL patients | P006 | 46 | Male | Diffuse Large B-Cell Lymphoma | CD20(+) | surgical resection | None | None |
| PCNSL patients | P007 | 74 | Female | Diffuse Large B-Cell Lymphoma | CD20(+) | stereotactic biopsy | None | None |
| PCNSL patients | P008 | 58 | Male | Diffuse Large B-Cell Lymphoma | CD20(+) | stereotactic biopsy | None | None |
| PCNSL patients | P009 | 78 | Male | Diffuse Large B-Cell Lymphoma | CD20(+) | stereotactic biopsy | None | None |
| PCNSL patients | P010 | 36 | Female | Diffuse Large B-Cell Lymphoma | CD20(+) | stereotactic biopsy | None | None |
| PCNSL patients | P011 | 56 | Male | Diffuse Large B-Cell Lymphoma | CD20(+) | surgical resection | None | None |
| PCNSL patients | P012 | 56 | Male | Diffuse Large B-Cell Lymphoma | CD20(+) | surgical resection | None | None |
| PCNSL patients | P013 | 72 | Male | Diffuse Large B-Cell Lymphoma | CD20(+) | stereotactic biopsy | None | None |
| PCNSL patients | P014 | 73 | Female | Diffuse Large B-Cell Lymphoma | CD20(+) | stereotactic biopsy | None | None |
| PCNSL patients | P015 | 64 | Male | Diffuse Large B-Cell Lymphoma | CD20(+) | stereotactic biopsy | None | None |
| PCNSL patients | P016 | 58 | Female | Diffuse Large B-Cell Lymphoma | CD20(+) | stereotactic biopsy | None | None |
| PCNSL patients | P017 | 52 | Female | Diffuse Large B-Cell Lymphoma | CD20(+) | stereotactic biopsy | None | None |
| PCNSL patients | P018 | 38 | Female | Diffuse Large B-Cell Lymphoma | CD20(+) | stereotactic biopsy | None | None |
| PCNSL patients | P019 | 64 | Male | Diffuse Large B-Cell Lymphoma | CD20(+) | stereotactic biopsy | None | None |
| PCNSL patients | P020 | 68 | Male | Diffuse Large B-Cell Lymphoma | CD20(+) | stereotactic biopsy | None | None |
| PCNSL patients | P021 | 46 | Male | Diffuse Large B-Cell Lymphoma | CD20(+) | stereotactic biopsy | None | None |
| PCNSL patients | P022 | 72 | Female | Diffuse Large B-Cell Lymphoma | CD20(+) | stereotactic biopsy | None | None |
| PCNSL patients | P023 | 37 | Female | Diffuse Large B-Cell Lymphoma | CD20(+) | stereotactic biopsy | None | None |
| PCNSL patients | P024 | 52 | Female | Diffuse Large B-Cell Lymphoma | CD20(+) | stereotactic biopsy | None | None |
| PCNSL patients | P025 | 69 | Female | Diffuse Large B-Cell Lymphoma | CD20(+) | surgical resection | None | None |
| PCNSL patients | P026 | 67 | Male | Diffuse Large B-Cell Lymphoma | CD20(+) | surgical resection | None | None |
| PCNSL patients | P027 | 62 | Female | Diffuse Large B-Cell Lymphoma | CD20(+) | stereotactic biopsy | None | None |
| PCNSL patients | P028 | 74 | Female | Diffuse Large B-Cell Lymphoma | CD20(+) | surgical resection | None | None |
| PCNSL patients | P029 | 36 | Male | Diffuse Large B-Cell Lymphoma | CD20(+) | surgical resection | None | None |
| PCNSL patients | P030 | 25 | Male | Diffuse Large B-Cell Lymphoma | CD20(+) | stereotactic biopsy | None | None |
| PCNSL patients | P031 | 65 | Male | Diffuse Large B-Cell Lymphoma | CD20(+) | stereotactic biopsy | None | None |
| PCNSL patients | P032 | 69 | Female | Diffuse Large B-Cell Lymphoma | CD20(+) | stereotactic biopsy | None | None |
| PCNSL patients | P033 | 73 | Male | Diffuse Large B-Cell Lymphoma | CD20(+) | stereotactic biopsy | None | None |
| Healthy Controls | C001 | 67 | Female | N/A | N/A | N/A | N/A | None |
| Healthy Controls | C002 | 62 | Male | N/A | N/A | N/A | N/A | None |
| Healthy Controls | C003 | 48 | Male | N/A | N/A | N/A | N/A | None |
| Healthy Controls | C004 | 68 | Male | N/A | N/A | N/A | N/A | None |
| Healthy Controls | C005 | 56 | Female | N/A | N/A | N/A | N/A | None |
| Healthy Controls | C006 | 46 | Male | N/A | N/A | N/A | N/A | None |
| Healthy Controls | C007 | 74 | Female | N/A | N/A | N/A | N/A | None |
| Healthy Controls | C008 | 58 | Male | N/A | N/A | N/A | N/A | None |
| Healthy Controls | C009 | 78 | Male | N/A | N/A | N/A | N/A | None |
| Healthy Controls | C010 | 36 | Male | N/A | N/A | N/A | N/A | None |
| Healthy Controls | C011 | 63 | Male | N/A | N/A | N/A | N/A | None |
| Healthy Controls | C012 | 48 | Female | N/A | N/A | N/A | N/A | None |
| Healthy Controls | C013 | 68 | Female | N/A | N/A | N/A | N/A | None |
| Healthy Controls | C014 | 56 | Female | N/A | N/A | N/A | N/A | None |
| Healthy Controls | C015 | 46 | Male | N/A | N/A | N/A | N/A | None |
| Healthy Controls | C016 | 74 | Male | N/A | N/A | N/A | N/A | None |
| Healthy Controls | C017 | 58 | Female | N/A | N/A | N/A | N/A | None |
| Healthy Controls | C018 | 78 | Female | N/A | N/A | N/A | N/A | None |
| Healthy Controls | C019 | 36 | Female | N/A | N/A | N/A | N/A | None |
| Healthy Controls | C020 | 56 | Female | N/A | N/A | N/A | N/A | None |
| Healthy Controls | C021 | 56 | Male | N/A | N/A | N/A | N/A | None |
| Healthy Controls | C022 | 72 | Male | N/A | N/A | N/A | N/A | None |
| Healthy Controls | C023 | 73 | Female | N/A | N/A | N/A | N/A | None |
| Healthy Controls | C024 | 64 | Male | N/A | N/A | N/A | N/A | None |
| Healthy Controls | C025 | 58 | Female | N/A | N/A | N/A | N/A | None |
| Healthy Controls | C026 | 52 | Male | N/A | N/A | N/A | N/A | None |
| Healthy Controls | C027 | 74 | Male | N/A | N/A | N/A | N/A | None |
| Healthy Controls | C028 | 64 | Male | N/A | N/A | N/A | N/A | None |
| Healthy Controls | C029 | 68 | Female | N/A | N/A | N/A | N/A | None |
| Healthy Controls | C030 | 69 | Male | N/A | N/A | N/A | N/A | None |
| Healthy Controls | C031 | 65 | Male | N/A | N/A | N/A | N/A | None |
| Healthy Controls | C032 | 72 | Female | N/A | N/A | N/A | N/A | None |
